# Congenital Cytomegalovirus Severity Definitions and Treatment Decisions around the World: A Systematic Scoping Review of the Literature

**DOI:** 10.3390/jcm13195997

**Published:** 2024-10-08

**Authors:** Giovanni Boscarino, Rossana Romano, Francesca Tegoni, Carlotta Iotti, Serafina Perrone, Susanna Esposito, Danilo Buonsenso

**Affiliations:** 1 Pediatric Clinic, University Hospital, Department of Medicine and Surgery, University of Parma, 43126 Parma, Italy; giovanni.boscarino@unipr.it (G.B.); rossana.romano@unipr.it (R.R.); francesca.tegoni@unipr.it (F.T.); carlotta.iotti@unipr.it (C.I.); 2 Neonatology Unit, University Hospital, Department of Medicine and Surgery, University of Parma, 43126 Parma, Italy; serafina.perrone@unipr.it; 3 Department of Woman and Child Health and Public Health, Fondazione Policlinico Universitario, Agostino Gemelli-IRCCS, 00168 Rome, Italy; danilobuonsenso@gmail.com; 4 Università Cattolica del Sacro Cuore, 00168 Rome, Italy

**Keywords:** cytomegalovirus, ganciclovir, valganciclovir, neurological outcome, hearing, neutropenia, neonates

## Abstract

Congenital cytomegalovirus (cCMV) is the most common cause of congenital infection and the leading cause of non-genetic sensorineural hearing loss in childhood. While treatment trials have been conducted in symptomatic children, defining asymptomatic infection can be complex. We performed a scoping review to understand how infection severity is defined and treated globally, as well as the various indications for initiating treatment. We conducted an electronic search of MEDLINE, EMBASE, Scopus, and the Cochrane Library, using combinations of the following terms: “newborn”, “baby”, “child”, “ganciclovir”, “valganciclovir”, and “cytomegalovirus” or “CMV”. We included eligible prospective and retrospective studies, case series, and randomized clinical trials (RCTs) published up to May 2024. A total of 26 studies were included, of which only 5 were RCTs. There was significant heterogeneity between studies. The most commonly considered criteria for symptomatic infection were microcephaly (23/24 studies), abnormal neuroimaging (22/24 studies), chorioretinitis/ocular impairment (21/24 studies), and hearing impairment (20/24 studies). Two studies also included asymptomatic newborns in their treatment protocols. Outcome measures varied widely, focusing either on different hearing assessments or neurocognitive issues. Our literature analysis revealed significant variability and heterogeneity in the definition of symptomatic cCMV infection and, consequently, in treatment approaches. A consensus on core outcomes and well-conducted RCTs are needed to establish treatment protocols for specific groups of newborns with varying manifestations of cCMV.

## 1. Introduction

Congenital cytomegalovirus (cCMV) is the most common cause of congenital infection in developed countries [1]. Although cCMV infection can sometimes present as a severe, systemic, life-threatening disseminated disease, such cases are relatively rare and can be treated with a six-week course of intravenous ganciclovir (GC), oral valganciclovir (VGC), or a combination of initial GC followed by VGC [2].

More commonly, cCMV infection is clinically asymptomatic. However, even in asymptomatic cases, cCMV infection can be associated with significant outcomes. It is recognized as the leading cause of non-genetic sensorineural hearing loss (SNHL) in childhood, regardless of infection severity [1,3]. Approximately 25% of SNHL cases in children up to four years old are attributed to cCMV [4]. Infants with symptomatic infections at birth are at higher risk of developing late-onset SNHL (LO-SNHL), with up to one-third affected, but asymptomatic newborns can also develop this condition during childhood [5,6]. Most cases of LO-SNHL occur before three years of age [6]. Additionally, increasing reports highlight neurocognitive problems in these children during their school years [7,8].

For these reasons, researchers focusing on cCMV and its outcomes have devoted significant attention to strategies that may prevent the long-term negative effects of the infection. A clinical trial involving symptomatic children with central nervous system (CNS) involvement and/or SNHL at birth demonstrated that antiviral treatment with ganciclovir or valganciclovir may prevent hearing deterioration [9,10]. However, evidence on the effectiveness of treatment in asymptomatic or mildly symptomatic cCMV cases remains limited [11]. To complicate matters further, the definition of asymptomatic or mild cCMV infection can vary, especially with the advent of increasingly advanced diagnostic tools. For example, a clinically healthy newborn, who years ago might have been classified as asymptomatically infected, could today fall under a different classification if a brain magnetic resonance imaging (MRI) were performed. This reflects the evolving criteria and the challenges associated with interpreting MRI findings in newborns. An isolated premature delivery of a small-for-gestational age (SGA) newborn may either be unrelated to maternal CMV infection or could indicate symptomatic infection. To date, these dilemmas have not been standardized or agreed upon internationally. As a result, therapeutic practices vary widely across the world, influenced by local guidelines and expert opinions. Some experts even recommend antiviral treatment for asymptomatic infections, despite the lack of strong supporting evidence [12,13].

To further investigate these issues, we conducted a scoping review of the literature to understand how cCMV infection severity is defined and how the infection is treated globally, as well as the various indications for initiating treatment. This study aims to provide researchers and policymakers with insights into the current variability in practice and to lay the groundwork for addressing uncertainties and standardizing clinical care and research priorities worldwide.

## 2. Materials and Methods

### 2.1. Search Strategy and Inclusion Criteria

The goal of our scoping review is to provide a comprehensive overview of the current literature on a broad neonatal topic. Initially, we aimed to conduct a meta-analysis; however, because of the heterogeneity in the published studies, a scoping review was deemed the most appropriate methodological approach. We followed the guidelines provided by the Preferred Reporting Items for Systematic Reviews and Meta-Analyses extension for Scoping Reviews (PRISMA-ScR) checklist [14].

The search strategy was proposed by a single author (GB) and discussed and approved by the entire team. It was based on a combination of the following terms: “newborn”, “baby”, “child”, “ganciclovir”, “valganciclovir”, and “cytomegalovirus” or “CMV”. We conducted the search using electronic databases (MEDLINE, EMBASE, Scopus, and the Cochrane Library) through customized search queries. Additionally, the references of the included articles were reviewed to identify other relevant studies. The references were regularly updated throughout the drafting of this review.

We included prospective and retrospective studies, case series, and randomized clinical trials (RCTs) published up to May 2024. Case reports and editorials were excluded, and only articles published in English were considered eligible. The main research questions of this scoping review were as follows:

How is symptomatic infection defined globally?

What are the global indications for treating cCMV infection?

What are the outcomes (hearing and neurological) and side effects of cCMV treatment?

### 2.2. Search Screening and Data Extraction

After conducting the search, the studies were exported to Rayyan for screening. The first screening to remove duplicates was carried out by one author (GB). Titles and abstracts of the studies identified through the search strategy were independently screened by four reviewers (RR, FT, CI) to identify studies for inclusion. Full texts of potentially eligible studies were retrieved and independently assessed for eligibility. Each researcher was blinded to the decisions of the other reviewers. Any disagreements regarding study eligibility were resolved through discussion, and in cases of unresolved disagreement, a third reviewer (GB) was consulted. Studies that did not meet the inclusion criteria were excluded.

Neurological outcomes were considered in terms of hearing and neurodevelopment. For each selected study, data were summarized in a table based on a pre-discussed data extraction form, which included details on the setting, authorship, year of publication, study design, treatment, definitions of cCMV and symptomatic infection, outcomes, and side effects. A separate table was created for RCTs. Additionally, treatment indications and the criteria for defining symptomatic cCMV were summarized in a specific data form.

## 3. Results

**Figure 1:**
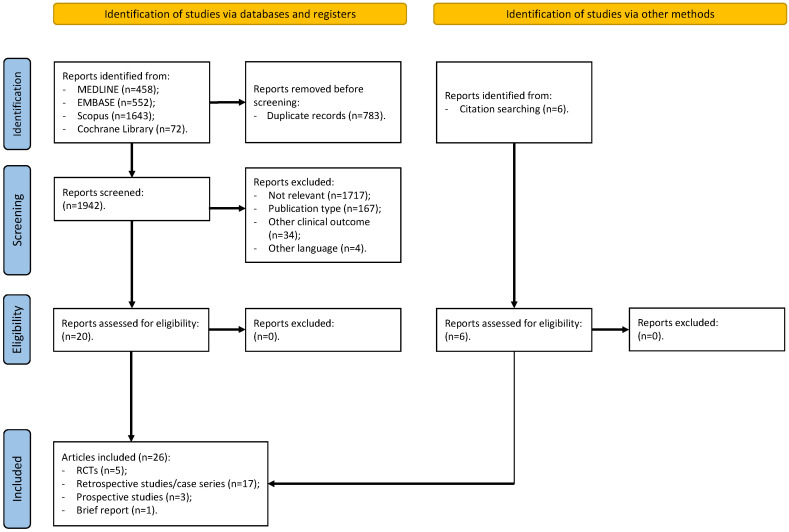
PRISMA flowchart. Figure legend: RCTs (randomized controlled trials).

Using the search terms described in the Methods Section, we identified 2725 articles (Figure 1). Following the screening process, we selected 20 research articles. After manually reviewing the reference lists of studies from previous stages (including systematic reviews and meta-analyses), we added six additional documents. Thus, as shown in Figure 1, the final step of this scoping review included 26 studies [9,10,12,15,16,17,18,19,20,21,22,23,24,25,26,27,28,29,30,31,32,33,34,35,36,37], consisting of five RCTs, 17 retrospective studies/case series, three prospective studies, and one brief report.

**Figure 2:**
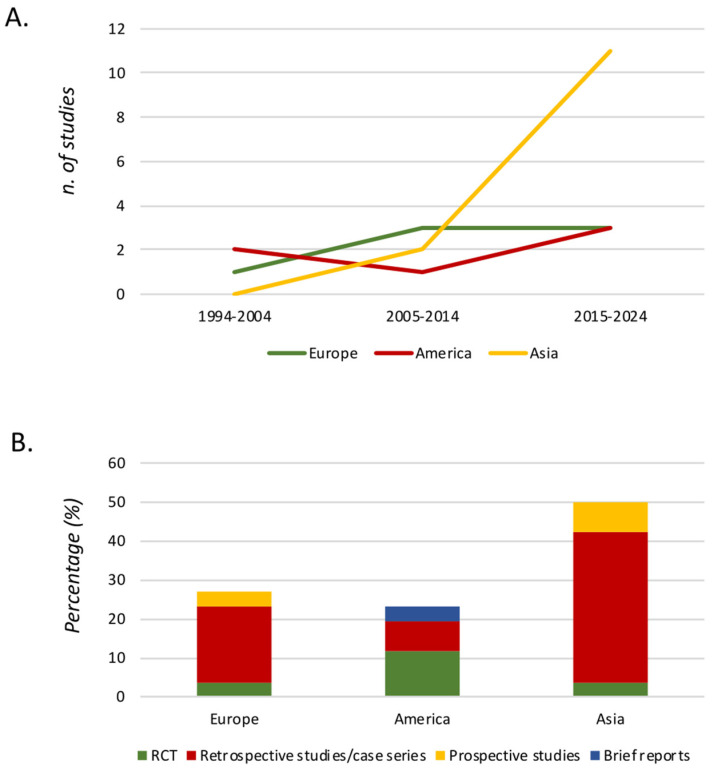
Graphical distribution of the studies according to year of publication. Figure legend. (**A**) Number of studies separated by continent and year of publication. (**B**) Typology of studies separated by continent. RCT (randomized controlled trial).

Figure 2 describes the distribution of the studies according to year of publication. It shows an increasing number of studies in the last decades, especially in Asia. Of these, seven (26.9%) were published in Europe, six (23.1%) were published in America, and the majority were published in Asia (13.50%), as shown in Figure 2.

Table 1 describes the criteria used to diagnose and define symptomatic cCMV in the enrolled studies, separated by continent.

In addition, Table 2 provides a graphical representation of the criteria used to define symptomatic cCMV in the studies. Two studies did not report criteria for symptomatic infection (Table 2). The most commonly considered criteria for symptomatic infection were microcephaly (23/24 studies), abnormal neuroimaging (22/24 studies), chorioretinitis/ocular impairment (21/24 studies), and hearing impairment (20/24 studies). The least frequently considered criteria were abnormal cerebrospinal fluid (CSF) findings (7/24 studies) and clinical neurological abnormalities (6/24 studies).

In Table 3, we summarize the treatment indications considered in the studies included in our scoping review. Two studies also included asymptomatic newborns in their treatment protocols. The treatment protocol was not well specified in three studies, while one study treated all newborns with severe neurological symptoms, and another study treated patients under compassionate use. The remaining 19 studies treated only symptomatic newborns.

The treatments used in the studies are summarized in Table 4. There was some heterogeneity between the studies. While the majority administered IV GC, comparing two different protocols in terms of dosage and timing of administration, some studies used IV GC followed by oral VGC or compared different protocols based on the dosage and duration of treatment with either GC/VGC or VGC alone. Additionally, we described the neurological and hearing outcomes, as well as the side effects, associated with the two treatment protocols used in the studies.

With the aim of conducting a sub-meta-analysis focused on RCTs, we described the outcomes (specifically hearing, neurodevelopment, and side effects) in Table 5. Hearing outcomes were assessed in three out of five RCTs, while two out of five RCTs evaluated long-term neurological outcomes. The most commonly studied side effect was neutropenia, reported in all five RCTs. However, we decided not to perform a meta-analysis because of the significant variability in treatment protocols, methodologies, and outcomes across the enrolled RCTs.

## 4. Discussion

In this scoping review, we analyzed the extensive literature on the treatment of cCMV, with a specific focus on understanding how infection severity is defined and how it is treated globally. We also examined the criteria used to decide when to initiate treatment. Our primary aim was to determine whether a standardized protocol for best practices could be established to promptly define symptomatic cCMV infection and treat affected newborns in a way that improves both short- and long-term outcomes. To our knowledge, this is the first systematic scoping review to address these perspectives with clinical implications.

Despite growing interest in this topic over the past decades, only five RCTs were published up to May 2024 [9,10,15,22,36], each involving different populations and outcomes. This represents a significant gap with important implications for daily clinical practice. It is known that neonates with asymptomatic cCMV infection (approximately 10% of cases) generally have better long-term outcomes compared with children with symptomatic infection [38,39]. Because of the potential toxicity of antiviral drugs, treatment is currently recommended only for symptomatic cases [40]. However, our scoping review revealed significant variability and heterogeneity in the definition of symptomatic cCMV infection, leading to differences in treatment indications across centers. Varying definitions of symptomatic disease can result in different interpretations of which infants might benefit most from antiviral treatment, thus influencing clinical decisions based on the perceived risks and benefits.

Moreover, asymptomatic cCMV can also cause SNHL during early childhood, with variable onset, progression, and severity [38,39]. Unfortunately, no clinical, laboratory, or imaging features are currently able to predict this risk reliably [39]. As a result, it remains unclear whether clinically well-appearing newborns with cCMV and isolated SNHL should receive antiviral treatment.

While most centers include the classic signs and symptoms of severe cCMV in their definition of symptomatic infection (such as microcephaly, chorioretinitis, cerebrospinal fluid abnormalities, and hematological abnormalities), there is greater variability when it comes to abnormal neuroimaging or hearing impairment. For instance, how should we classify a clinically well-appearing cCMV newborn with mild audiological impairment or isolated white matter abnormalities? Is this a symptomatic or asymptomatic infection, and should such cases be treated? Historically, clinical symptoms alone were used to define symptomatic infection. However, with the increasing availability of newborn hearing screening and advanced imaging (including ultrasound and the growing use of MRI), there is ongoing debate about whether isolated abnormalities should be considered part of symptomatic infection and treated accordingly.

Chung et al. investigated the benefits of antiviral treatment for children with isolated hearing loss and clinically inapparent cCMV in a nonrandomized trial published in May 2024 [41]. They found that children with inapparent cCMV and hearing loss who were treated with VGC experienced less hearing deterioration at 18 to 22 months of age compared with the control group [41]. These results led to the implementation (edition 2024) of recent European guidelines for the management of cCMV infection. This update suggests the benefit of treatment as soon as possible and before 1 to 3 months of age also for infants with isolated SNHL [42]. However, these points are critical, as SNHL is the most common long-term outcome of cCMV infection, and recent studies suggest that even isolated brain MRI abnormalities might be predictive of this outcome. A recent retrospective, single-center observational study, which included 225 patients with cCMV who underwent neonatal brain MRI with diffusion-weighted imaging between 2007 and 2020, found that the general white matter apparent diffusion coefficient was significantly higher in patients with neonatal hearing loss and cognitive or motor impairment (*p* <  0.05) [43]. In another study, abnormal white matter was associated with neonatal hearing loss and lower motor scores, with a tendency towards impaired cognitive development [44]. Similarly, a smaller Spanish study of 36 patients reported that MRI brain abnormalities were more frequent in newborns with SNHL (11 of the 36 patients had MRI abnormalities and SNHL, *p* = 0.004) [45].

In a larger Spanish study involving 160 infants with cCMV (103 symptomatic), temporal-pole white matter abnormalities, rather than the extent of white matter abnormalities, were associated with moderate/severe disability (OR 7.8; 1.4–42.8), specifically severe SNHL or SNHL combined with other moderate/severe disabilities (OR 16.2; 1.8–144.9) [46]. A smaller series of 17 patients in the United States showed that most infants whose CMV infections were identified after failing newborn hearing screening had abnormal brain MRIs [47]. Conversely, a small study from the Republic of Korea, involving 31 patients, did not find any association between MRI findings and SNHL [48].

While these findings are noteworthy, they raise the question of whether all newborns with cCMV, including those with clinically asymptomatic infections, should undergo neonatal MRI. Although MRI shows promise as a prognostic tool, routine use may not be feasible in all settings because of its high cost (both for the test itself and the extended hospital stay required, even for healthy newborns), as well as the potential need for sedation, making its widespread application challenging. Additionally, interpreting brain MRI findings in newborns can be difficult, as some results may reflect physiological delays in maturation rather than specific abnormalities associated with cCMV [49]. Lastly, whether children with a clinically asymptomatic infection but with brain MRI abnormalities should be treated with antivirals still needs to be addressed. However, as shown in Table 3 of our study, some authors use brain MRI abnormalities as an indication of treatment; however, this is not specifically mentioned in any guidelines, nor is there evidence of any potential benefits.

In fact, trials or observational studies that have treated patients historically used other definitions as inclusion criteria for treatment; nevertheless, it seems that clinicians translate those historical findings to expand indications for treatment. Nowadays, GC, and its oral pro-drug VGC, are the two antiviral drugs administered to treat symptomatic cCMV. Other potential drugs for cCMV disease are Foscarnet and Cidofovir, but these are not routinely administered. Myelosuppression, especially neutropenia, is the most frequent side effects associated with GC and VGC [50]. Foscarnet and Cidofovir result in renal toxicity and electrolyte imbalances [50]. To the best of our knowledge, there are no RCTs that evaluated the treatment with Foscarnet or Cidofovir for newborns with cCMV infection. In addition, there is an ongoing phase 1 trial that is evaluating the role of oral Letermovir as a potential alternative to VCG for symptomatic cCMV infection (NCT06118515, ClinicalTrials.gov).

Nigro et al. conducted an RCT comparing infants with symptomatic cCMV treated with two different IV ganciclovir (GC) protocols (Group A: 5 mg/kg twice daily for 2 weeks vs. Group B: 7.5 mg/kg twice daily for 2 weeks, followed by 10 mg/kg three times weekly for 3 months) [15]. The authors did not assess hearing or neurological outcomes and found no statistical difference in terms of neutropenia between the groups. However, the study had a very small sample size (six infants in each group).

In 2003, Kimberlin et al. enrolled 100 neonates (≥32 weeks and ≥1200 g at birth) with symptomatic cCMV involving the CNS, defined by microcephaly, intracranial calcifications, abnormal CSF for age, chorioretinitis, and/or hearing deficits [9]. They compared two groups, one treated with IV GC (6 mg/kg every 12 h for 6 weeks) and the other untreated. The authors found that GC therapy prevented hearing deterioration at 6 months and might also prevent deterioration after 1 year, despite two-thirds of the treated newborns developing neutropenia during therapy.

In a follow-up study, Oliver et al. evaluated neurological outcomes using the Denver II scale at 6 weeks, 6 months, and 12 months [22]. Their results confirmed previous findings on the benefits of GC therapy for symptomatic cCMV, including its toxicity, particularly neutropenia. In 2015, the same authors evaluated two different oral valganciclovir (VGC) treatment protocols in the same population of neonates (≥32 weeks and ≥1200 g at birth). Group A received 16 mg/kg of oral VGC every 12 h for 6 months, while Group B received 16 mg/kg of oral VGC every 12 h for 6 weeks, followed by a placebo up to 6 months [10]. They found that treating symptomatic cCMV with VGC for 6 months, compared with 6 weeks, did not improve hearing in the short term but appeared to improve hearing and developmental outcomes after 12 months, as measured by the Bayley Scales of Infant and Toddler Development, third edition. The risk of neutropenia was similar between the two groups after 6 weeks and up to 6 months, suggesting that the highest risk occurs during the first 6 weeks of treatment.

The most recent RCT was conducted by Yang et al. [36]. They compared symptomatic newborns treated with IV GC (6 mg/kg every 12 h for 6 weeks) and oral VGC (16 mg/kg every 12 h for 6 weeks). No differences were found in hearing outcomes or side effects between the two groups, although the oral route of VGC was more acceptable for neonates.

Our study should be interpreted with some limitations. This is a scoping review that aims to better describe the definition of cCMV and evaluate the criteria for the treatment of cCMV across the world and the best therapy weighing the outcomes versus the side effects. We decided not to perform a meta-analysis because of the heterogeneity in the published studies in terms of the criteria for the diagnosis and treatment of cCMV (Table 2 and Table 3). We systematically collected evidence with a robust research strategy and after a deep evaluation and discussion between the authors. We selected articles published in English up to May 2024; thus, it is possible that some gray literature or articles published after May 2024 were analyzed. Despite its limitations, the current literature does not allow us to determine the best antiviral therapy for cCMV for several reasons. First, very few randomized controlled trials (RCTs) have been published, and these are the best type of study for evaluating treatments. It is not possible to determine the most effective drug therapy based on observational or retrospective studies. A key finding of our review is that the included studies used different outcomes, measured with different methods, neurological scales, or in varying populations. Even when assessing audiological outcomes, which should theoretically be the easiest to measure, different authors employed various methods at different timepoints. Neurological clinical signs were considered for the definition of symptomatic patients in only 6 studies [12,15,18,20,21,37]. Additionally, treatment protocols differed between studies in terms of dosage and duration of therapy.

One of the most significant limitations is that the published RCTs did not include asymptomatic newborns. To our knowledge, only two retrospective studies included asymptomatic newborns with congenital infection in their treatment protocols [12,16]. Lackner et al. treated asymptomatic cCMV with IV ganciclovir (10 mg/kg for 21 days) [16], finding improved outcomes for treated infants but no differences in long-term neurological outcomes. Turriziani Colonna et al. evaluated the long-term audiological, visual, neurocognitive, and behavioral outcomes in both symptomatic and asymptomatic cCMV patients treated with oral valganciclovir (VGC) [12], showing that both groups developed long-term sequelae. However, their study lacked a control group. While these studies suggest that asymptomatic cCMV might benefit from antiviral treatment, they do not provide conclusive evidence, as they were retrospective with small sample sizes. Given the differences in pharmacological approaches and study populations (asymptomatic vs. symptomatic, with varying definitions of symptomatic infections), we believe that the available data do not meet the minimum criteria necessary to perform a meta-analysis on this outcome.

Further complicating treatment decisions, valacyclovir was recently approved for use in pregnant women with CMV infection to prevent vertical transmission to the newborn [51]. A recent meta-analysis of three RCTs, involving 527 women, demonstrated that oral valacyclovir reduces the risk of vertical transmission (adjusted OR 0.34, 95% CI, 0.18–0.61), whether administered during the periconceptional period (adjusted OR 0.34; 95% CI, 0.12–0.96) or the first trimester of pregnancy (adjusted OR 0.35; 95% CI, 0.16–0.76) [51]. This suggests that in the future, an increasing number of newborns with cCMV will be born to mothers treated with valacyclovir during pregnancy, raising questions about whether the criteria used during the pre-valacyclovir era will still apply. For instance, will these children be at risk for late-onset SNHL? If they are asymptomatic, should they still receive VGC despite their in utero exposure to valacyclovir? Many questions remain unanswered.

As we gain more experience with VGC, and as most side effects appear to be minor and transient, as shown in Table 4, there is potential to expand the use of VGC to asymptomatic newborns with isolated abnormalities. However, to achieve this, we need a new generation of RCTs that are appropriately designed, using rigorous clinical definitions of infection severity and standardized outcome measures. A key priority for scientific societies involved with cCMV should be the development of a core outcome set for cCMV trials, ensuring that data from different centers can be combined and compared.

## 5. Conclusions

Our scoping review showed that interest in studying cCMV treatment is growing globally. However, we identified significant variability and heterogeneity in the definition of symptomatic cCMV infection, leading to differences in treatment decisions. Moreover, therapeutic protocols varied between studies, and outcomes (such as hearing function) were measured using different methods, limiting the ability to compare and interpret results. Thus, there is an urgent need for consensus on defining symptomatic and asymptomatic cCMV infection, using the most advanced methodologies available (e.g., brain MRI in certain settings). Additionally, a core outcome set should be developed, with pre-specified outcomes that can be used across research centers to ensure comparability. These definitions will be crucial for designing new therapeutic RCTs to improve the short- and long-term outcomes for children with cCMV.

## Figures and Tables

**Table 1:** Diagnosis and definition of symptomatic cCMV used in the enrolled studies, separated by continent.

Continent	Setting	Reference	Type of Study	Population	Diagnosis of cCMV	Symptomatic cCMV
*Europe*	Italy–Germany	Nigro 1994 [15]	RCT	Infants with symptomatic cCMV	Virus isolation, identified by its typical cytopathic effect, and/or detection of CMV-DNA within the first 2 wks of life plus CMV-specific IgM or IgA or significant levels of IgG (i.e., values one and one-half times higher in infants than in their mothers).	Microcephaly, chorioretinitis, hypotonia, hypertonia, seizures, hepatosplenomegaly.
	Austria	Lackner 2009 [16]	Retrospective cohort study	Children with documented, asymptomatic cCMV infection	Detection of CMV immunoglobulin M in maternal serum or newborn umbilical cord vein blood, and identified by isolation of the virus in urine during the first postnatal wk.	Microcephaly, hydrocephaly, ventriculomegaly, chorioretinitis or other ophthalmological symptoms, hepato-splenomegaly, thrombocytopenia, neutropenia, anemia, jaundice, or hearing disorders.
	Belgium	Foulon 2012 [17]	Prospective study	Children with a proven cCMV infection with a minimum length of follow up of 18 months	Positive urine culture performed within seven days after birth.	Hepatosplenomegaly, petechiae, jaundice, or microcephaly.
	Spain	del Rosal 2012 [18]	Retrospective case series	cCMV with CNS involvement	CMV-PCR in dried blood spots. Cases in which dried blood spots were not available were considered as having suspected congenital CMV infection provided they fulfilled all the following conditions: (1) positive urine CMV-PCR, (2) suggestive clinical and neuroimaging findings, and (3) exclusion of other congenital infections and neurological disorders.	CNS involvement: microcephaly, chorioretinitis, abnormal visual or auditory evoked responses, neurologic signs, or abnormal neuroimaging findings.
	Italy	Turriziani Colonna 2020 [12]	Retrospective study	Treated cCMV infection (both symptomatic and asymptomatic)	CMV-DNA found in blood or urine using real-time PCR in the first 3 wks of life.	Petechiae, hepatomegaly, splenomegaly, abnormalities in blood chemistry (thrombocytopenia <100,000/μL, anemia, leukopenia, elevation of liver enzymes, conjugated hyperbilirubinemia), SGA < −2 DS status, neurologic and/or ophthalmologic examination anomalies, microcephaly, convulsions, neuroradiological abnormalities related to CMV infection, abnormalities in ABR.
	Poland	Jedlińska-Pijanowska 2020 [19]	Retrospective study	Symptomatic treated cCMV	Positive real-time PCR results for HCMV- DNA in urine before or on the 21st day of life.	CNS abnormalities: microcephaly, abnormal neuroimaging (intracerebral calcifications, intra/paraventricular cysts, ventriculomegaly), sensorineural hearing loss, chorioretinitis, and/or a minimum of three hepatobiliary and reticuloendothelial system disorders (including hepatomegaly, splenomegaly, petechiae, thrombocytopenia, neutropenia, hepatitis, and cholestasis).
	Italy	Venturini 2023 [20]	Retrospective study	cCMV infection	CMV-DNA by real-time PCR-positive urine, saliva, blood, or CSF samples collected in the first 3 wks of life, or if a positive result was demonstrated on dried blood spot obtained on 3–5 days of life.	Mildly symptomatic, subjects with one to two mild and transient manifestations. Moderately to severely symptomatic: chorioretinitis, sensorineural hearing loss, and/or central nervous system manifestations, sometimes associated with hematologic or other transient manifestation.
*America*	United States	Kimberlin 2003 [9]	RCT	Neonates (≥32 wks and ≥1200 g at birth) with symptomatic cCMV involving the CNS	Confirmed isolation of CMV from a urine specimen in the first month of life.	CNS disease (microcephaly, intracranial calcifications, abnormal cerebrospinal fluid for age, chorioretinitis, hearing deficits).
	United States	Michaels 2003 [21]	Retrospective chart review	Children < 1 year of age who were identified as having symptomatic cCMV infection with CNS involvement, hearing impairment, or both treated with GC	Viral urine culture.	CNS involvement, hearing impairment or both, hepatosplenomegaly, thrombocytopenia, petechiae/purpura.
	United States	Oliver 2009 [22]	RCT	Neonates with symptomatic cCMV infection, ≥32 wks, ≥1200 g	Isolation of CMV from a urine specimen obtained prior to study enrollment and within the first month of life.	CNS involvement: microcephaly, intracranial calcifications, abnormal CSF for age, chorioretinitis, or hearing loss.
	United states	Kimberlin 2015 [10]	RCT	Neonates (≥32 wks or ≥30 days of life and ≥1800 g at the initiation of therapy) with symptomatic cCMV	Urine or throat-swab specimens by means of culture, shell vial culture, or PCR.	Thrombocytopenia, petechiae, hepatomegaly, splenomegaly, intrauterine growth restriction, hepatitis, or CNS involvement such as microcephaly, intracranial calcifications, abnormal cerebrospinal fluid indexes, chorioretinitis, sensorineural hearing loss, or the detection of CMV DNA in cerebrospinal fluid.
	United States	McCrary 2019 [23]	Retrospective chart review	Symptomatic cCMV patients treated with VGC	Urine or saliva specimens within the first 3 wks of life, or dried blood spot testing as part of the metabolic screening during the first days of life.	Thrombocytopenia, petechiae, hepatomegaly, splenomegaly, intrauterine growth restriction, hepatitis, microcephaly, intracranial calcifications, abnormal CSF indexes, or chorioretinitis.
	United States	Leung 2022 [24]	Brief report	cCMV diagnosis within 45 days of life	Not reported.	Not reported.
*Asia*	Israel	Amir 2010 [25]	Retrospective case-series	cCMV treated with GC/VGC	Positive urine culture for CMV (shell vial method) at age up to 2 weeks.	CNS involvement: microcephaly; hearing impairment detected by ABR; chorioretinitis; abnormal findings on brain US (calcification, periventricular hyperechosity, ventricular dilatation, pseudocyst, and lenticular striated vasculopathy).
	Israel	Amir 2013 [26]	Retrospective study	Treated cCMV	Positive urine culture (shell vial) obtained during the first 2 wks of life.	Abnormal blood count, liver and kidney function tests, fundoscopy (performed by a pediatric ophthalmologist), and brain ultrasound over the anterior and posterior fontanel. Not appropriate birth weight for gestational age.
	Israel	Bilavsky 2015 [27]	Retrospective study	cCMV Group one: no hearing impairment at birth who, not treated with GC/VGC Group two: LSV and no hearing impairment, treated with GC/VGC Group three: LSV and hearing loss, treated with GC/VGC Group four: asymptomatic cCMV, not treated with GC/VGC	Positive urine culture, using a shell vial assay, obtained during the first two wks of life.	Microcephaly, hearing impairment detected by the brainstem evoked response audiometry test, chorioretinitis, and abnormal findings on brain ultrasound including calcifications, periventricular hyperechosity, ventricular dilatation, and pseudocysts.
	Israel	Bilavsky 2016 [28]	Retrospective study	Symptomatic cCMV, who started antiviral treatment during the first 4 wks of life	Positive urine culture (shell vial) or PCR taken during the first 2 wks of life.	CMS involvement: microcephaly, hearing impairment detected by the brainstem evoked audiometry, chorioretinitis, or abnormal findings on brain US compatible with cCMV (e.g., calcifications, periventricuar hyperechosity, ventricular dilatation, pseudocysts, and lenticulostriate vasculopathy).
	Japan	Nishida 2016 [29]	Prospective study	cCMV	Urine samples collected from newborns onto filter paper within 1 wk after birth. Liquid urine samples were obtained from CMV-positive newborns, and the CMV-DNA copy number was determined by real-time quantitative PCR.	Microcephaly, hepatosplenomegaly/hepatitis (serum alanine aminotransferase level >100 U/L), thrombocytopenia (platelet count < 1 × 10^5^/lL), brain image abnormality (intracranial calcifications, ventricular dilation, white matter abnormality, and cortical dysplasia), CMV associated retinopathy, or abnormal ABR.
	Japan	Koyano 2018 [30]	Retrospective study	cCMV	Urine filter system.	Microcephaly, chorioretinitis, hearing disability, or a combination of petechiae, hepatosplenomegaly, and jaundice, and/or abnormalities on brain imaging, such as intracranial calcification and ventricular enlargement. Patients with only intrauterine growth restriction or with any single, mild manifestation were not included in this category.
	Israel	Pasternak 2018 [31]	Retrospective study	cCMV and pathological brainstem evoked audiometry. Symptomatic infants were excluded	Diagnosed by a positive urine culture (shell vial) or polymerase chain reaction on specimens taken during the first 2 wks of life.	CNS involvement with microcephaly and/or intracranial calcifications on US of the brain and/or chorioretinitis and/or the presence of one or more of a non-CNS manifestation such as thrombocytopenia, petechial rash, hepatomegaly, splenomegaly, intrauterine growth retardation, or hepatitis.
	Israel	Ziv 2018 [32]	Retrospective study	cCMV	Positive urine culture (shell vial) or PCR performed during the first 2 wks of life.	CNS involvement with microcephaly, intracranial calcifications, periventricular hyperechosity, ventricular dilatation, pseudocyst, or LSV; sensorineural hearing loss detected by brainstem evoked response audiometry, presenting with mild to severe hearing loss; chorioretinitis; thrombocytopenia, petechia, hepatomegaly, splenomegaly, intrauterine growth restriction, or hepatitis.
	Japan	Ohyama 2019 [33]	Prospective study	Symptomatic cCMV	Diagnosed by a positive result in urine or the presence of clinical symptoms and findings of cCMV infection; a positive result of real-time quantitative PCR for CMV DNA in the urine as a confirmed diagnosis, made within 3 weeks of birth.	Microcephaly, hepatosplenomegaly (diagnosed with abdominal US or X-ray images)/hepatitis (ALT ≥ 100 U/L), thrombocytopenia (PLT < 100,000/μL), brain imaging abnormalities (intracranial calcification, ventricular dilatation, cortical dysplasia, or white matter injury), eye complications such as retinal choroiditis (diagnosed with fundoscopy), and abnormal ABR.
	Israel	Dorfman 2019 [34]	Retrospective study	cCMV who started antiviral treatment > 4 wks of life	Positive urine culture (shell vial) or urine PCR taken during the first 2 wks of life.	CNS (microcephaly, hearing impairment detected by brainstem evoked response audiometry, chorioretinitis, abnormal findings on brain US compatible with cCMV). Non-CNS manifestation (thrombocytopenia, petechiae, hepatomegaly, splenomegaly, intrauterine growth retardation, and hepatitis).
	Japan	Suganuma 2020 [35]	Retrospective study	cCMV infection	CMV DNA in urine or blood within 3 wks of life. For those who were more than 3 wks old at the first visit, a diagnosis was made by detecting CMV DNA in the dried umbilical cord, which was preserved according to traditional customs that respect the bond between mother and child in Japan, or preserved dried blood spots on filter paper that were collected for newborn mass congenital metabolic disorder screening.	Not reported.
	China	Yang 2021 [36]	RCT	Newborns with cCMV infection	Real-time PCR of saliva, urine, or both within the first 3 wks of life, with saliva as the preferred sample (Fifth International Congenital CMV Conference in 2015).	Over three of the following moderate to severe symptoms proposed in the Fifth International Congenital CMV Conference in 2015: thrombocytopenia, petechiae, hepatomegaly, splenomegaly, intrauterine growth restriction, hepatitis (raised transaminases or bilirubin), or CNS involvement such as microcephaly, radiographic abnormalities consistent with cytomegalovirus central nervous system disease (ventriculomegaly, intracerebral calcifications, periventricular echogenicity, cortical or cerebellar malformations), abnormal cerebrospinal fluid indices for age, chorioretinitis, sensorineural hearing loss, or the detection of cytomegalovirus DNA in cerebrospinal fluid.
	Thailand	Wongwathanavikrom 2021 [37]	Retrospective study	Treated cCMV infection	Microbiologic confirmation defined as a positive urine CMV isolation test within 3 wks of life, or the presence of clinical and radiologic features compatible with cCMV infection plus positive CMV serology in patients older than 3 wks of age.	The following clinical manifestations of symptomatic cCMV were reported in the study population: SGA, microcephaly, hepatobiliary, anemia, thrombocytopenia, rash, congenital heart problems, hypoglycemia, RDS, sepsis, neurology manifestation, NEC.

Table legend. cCMV (congenital cytomegalovirus infection); CNS (central nervous system); PCR (polymerase chain reaction); wks (weeks); SGA (small-for-gestational age); ABR (auditory brainstem response); DNA (deoxyribonucleic acid); CFS (cerebrospinal fluid); RCT (randomized controlled trial); RDS (respiratory distress syndrome); NEC (necrotizing enterocolitis); GC (ganciclovir); VGC (valganciclovir); LSV (lenticulostriated vasculopathy); US (ultrasonography); ALT (alanine aminotransferase); PLT (platelet).

**Table 2:** Criteria used to define symptomatic cCMV in the enrolled studies, separated by continent.

Continent	Setting	Reference	*Microcephaly*	*Chorioretinitis/Ocular Impairment*	*Hearing Impairment*	*Abnormal Neuroimaging*	*Neurological Abnormal Signs*	*Abnormal CSF*	*Petechiae*	*Hepatomegaly*	*Splenomegaly*	*IUGR or SGA*	*Thrombocytopenia*	*Hepatitis/Cholestasis §*	*Other Hematologic Findings*
*Europe*	Italy–Germany	Nigro 1994 [15]													
	Austria	Lackner 2009 [16]													
	Belgium	Foulon 2012 [17]													
	Spain	del Rosal 2012 [18]													
	Italy	Turriziani Colonna 2020 [12]													
	Poland	Jedlińska-Pijanowska 2020 [19]													
	Italy	Venturini 2023 [20]													
*America*	United States	Kimberlin 2003 [9]													
	United States	Michaels 2003 [21]													
	United States	Oliver 2009 [22]													
	United states	Kimberlin 2015 [10]													
	United States	McCrary 2019 [23]													
	United States	Leung 2022 [24]													
*Asia*	Israel	Amir 2010 [25]													
	Israel	Amir 2013 [26]													
	Israel	Bilavsky 2015 [27]													
	Israel	Bilavsky 2016 [28]													
	Japan	Nishida 2016 [29]													
	Japan	Koyano 2018 [30]													
	Israel	Pasternak 2018 [31] *													
	Israel	Ziv 2018 [32]													
	Japan	Ohyama 2019 [33]													
	Israel	Dorfman 2019 [34]													
	Japan	Suganuma 2020 [35]													
	China	Yang 2021 [36]													
	Thailand	Wongwathanavikrom 2021 [37]													
				Legend											
					Criterion included										
					Criterion not included										
					The study did not report the criteria for symptomatic infection										

Table legend. cCMV (congenital cytomegalovirus infection); CFS (cerebrospinal fluid); IUGR (intrauterine growth restriction); SGA (small-for-gestational age). § or raised transaminases or bilirubin. * Authors included only newborns with isolated hearing loss, all newborns that presented other symptoms related to cCMV were excluded.

**Table 3:** Indication for treatment used in the enrolled studies.

Reference	Asymptomatic	CNS Involvement						Thrombocytopenia	Petechiae	Hepatomegaly or Splenomegaly	IUGR or SGA	Other
		Microcephaly	Chorioretinitis	Hearing Impairment	Abnormal Neuroimaging	Neurological Abnormal Signs	Abnormal CSF					
Nigro 1994 [15]		X	X		Calcifications	X	X			X		
Kimberlin 2003 [9]		X	X	X	Intracranial calcifications		X					
Michaels 2003 [21]		X		X	Intracranial calcifications	X		X	X	X		
Lackner 2009 [16]	X											
Oliver 2009 [22]		X	X	X	Intracranial calcifications		X					
Amir 2010 [25]		X	X	X	Calcification, periventricular hyperechosity, ventricular dilatation, pseudocyst, LSV							
Foulon 2012 [17]	Severe neurological symptoms, not well described											
del Rosal 2012 [18]	Not well specified. Patients were treated under compassionate use											
Amir 2013 [26]				X								
Kimberlin 2015 [10]		X	X	X	Intracranial calcifications		X	X	X	X	IUGR	Hepatitis
Bilavsky 2015 [27]		X	X	X	Calcification, periventricular hyperechosity, ventricular dilatation, pseudocyst, LSV							
Bilavsky 2016 [28]		X	X	X	Calcifications, periventricular hyperechosity, ventricular dilatation, pseudocysts, LSV							
Nishida 2016 [29]		X	X	X	Intracranial calcifications, ventricular dilation, white matter abnormality, cortical dysplasia			X		X		
Koyano 2018 [30]		X	X	X	intracranial calcification, ventricular enlargement				X	X		Jaundice
Pasternak 2018 [31]				X								
Ziv 2018 [32]		X	X	X	Intracranial calcifications, periventricular hyperechosuty, ventricular dilatation, pseudocysts, LSV			X	X	X	IUGR	Hepatitis
McCrary 2019 [23]		X	X		Intracranial calcifications		X	X	X	X	IUGR	Hepatitis
Ohyama 2019 [33]		X	X	X	Intracranial calcification, ventricular dilatation, cortical dysplasia or white matter injury			X		X		Hepatitis (ALT ≥ 100 U/L)
Dorfman 2019 [34]		X	X	X	Calcifications, periventricular hyperechosity, ventricular dilatation, pseudocysts, LSV			X	X	X	IUGR	Hepatitis
Suganuma 2020 [35]	Not well specified											
Jedlińska-Pijanowska 2020 [19]		X	X	X	Intracerebral calcifications, intra/paraventricular cysts, ventriculomegaly			X	X	X		Neutropenia, hepatitis, cholestasis
Turriziani Colonna 2020 [12]	X	X	X	X	Calcifications, cystic periventricular leukomalacia, subependymal pseudocysts, germinolytic cyst, white matter anomalies, cortical atrophy, migration disorders, cerebellar hypoplasia, LSV	X		X	X	X	SGA	Anemia, leukopenia, elevation of liver enzymes, conjugated hyperbiirubinemia
Yang 2021 [36]		X	X	X	Ventriculomegaly, intracerebral calcifications, periventricular echogenicity, cortical or cerebellar malformations		X	X	X	X	IUGR	Hepatitis (raised transaminases or bilirubin)
Wongwathanavikrom 2021 [37]	Not well specified											
Leung 2022 [24]	Not well specified											
Venturini 2023 [20]		X	X	X	Ventriculomegaly, intracerebral calcifications, periventricular echogenicity, cortical or cerebellar malformations	X	X	X	X	X	IUGR	Hepatitis (raised transaminases or bilirubin)

Table Legend. CNS (central nervous system); IUGR (intrauterine growth restriction); SGA (small-for-gestational age); LSV (lenticulostriated vasculopathy).

**Table 4:** Hearing outcomes, neurological outcomes, and side effects reported in the enrolled studies.

Reference	Group A (GA)	Group B (GB)	Hearing Outcome			Neurological Outcome			Side Effects
			Definition of Hearing Loss	Time Point	Results	NDV Scales	Time Point	Results	
Nigro 1994 [15]	GC: 5 mg/kg twice daily for 2 weeks IV.	GC: 7.5 mg/kg twice daily for 2 weeks, and 10 mg/kg three times weekly for 3 months IV.	NE			NE			Increased liver enzyme activity: two out of six vs. zero out of six; High bilirubin level (>2.5 mg/dL): one out of two vs. zero out of six; Low platelet count (<90,000/ram 3): one out of six vs. zero out of six; Low hemoglobin value (<9 gm/dL): two out of six vs. zero out of six; Neutropenia (<1000 cells/mm^3^): zero out of six vs. one out of six.
Kimberlin 2003 [9]	GC (6 mg/kg per dose IV every 12 h for 6 wks).	No treatment.	Audiologic assessments were made by BSER. Mild hearing loss 21 to 45 dB thresholds, moderate hearing loss 46 to 70 dB thresholds, and severe hearing loss ≥71 dB thresholds.	BSER were made at study entry, 6 wks, 6 months, 1 year, and 2 years.	-A total of 0/25 in GA vs. 7/17 in GB (*p* < 0.01) had worsening in hearing at 6 months. -A total of 5/24 in GA vs. 13/19 in GB (*p* < 0.01) had worsening of hearing after 1 year.	NE			-GA had more rapid resolution of ALT abnormalities compared with GB (median time to ALT normalization, 19 days versus 66 days, respectively) (*p* = 0.03). -Neutropenia: 29/46 in GA vs. 9/43 in GB (*p* < 0.01). -Thrombocytopenia, organomegaly: no differences.
Michaels 2003 [21]	IV GC (10 mg/kg/day) started between 7 days and 11 months of age, decreased to 5 mg/kg/day after 2 to 4 weeks. Subsequent GC was administered OR (550 mg/m^2^/dose three times a day) for a median of 10 months.		ABR or developmentally appropriate behavioral hearing tests such as visual reinforcement audiometry for obtaining pure tone thresholds. Mild hearing loss: 21 to 40 dBHL; moderate hearing loss: 41 to 55 dBHL; moderately severe hearing loss: 56 to 70 dBHL; severe hearing loss more than 70 dBHL.	Not well reported	From baseline to most recent test: 2/18 ears improved; 16/18 ears showed no change.	Physical examination (high-pitched cry, abnormal tone, head lag or contractures or with formal developmental testing appropriate for age).	Not well reported	Abnormal development at follow-up: three out of nine.	Six out of nine had bacterial infections. One out of nine had neutropenia (<500 × 10^6^ cells/L).
Lackner 2009 [16]	IV GC within the first 10 days of life, 10 mg/kg for 21 days. If any signs of toxicity occurred (such as leucopoenia or diarrhea), the dosage was lowered to 5 mg/kg; therapy was stopped if side effects did not resolve.	No treatment.	Median sensorineural decrease in hearing of ≥10 dBHL at low (125 to 1000 Hz), middle (1000 to 4000 Hz), or high (4000 to 16,000 Hz) frequencies and was graded as mild (25 to ≤40 dBHL), moderate (41 to ≤65 dBHL), severe (66 to ≤96 dBHL), or profound (>96 dbHL).	Ear microscopy, middle-ear impedance tests, and behavioural observation audiometry until the age of four years; pure tone audiometry was used for older children (up to 10 years).	Hearing loss at follow-up: 0/10 GA vs. two out of eight GB.	Physical examination (speech and general development).	Up to 10 years.	Normal development in all children.	Moderate neutropenia: 2/12 in GA.
Oliver 2009 [22]	IV GC 12 mg/kg/die in two doses for 6 wks.	No treatment.	NE			(1) Denver II development test (Personal/Social, Fine Motor, Gross Motor and Language). (2) Denver II development test excluding the Language category.	At 6 and 12 months.	Total delays at 6 months in GA: 4.46 ± 0.74 and GB: 7.51 ± 1.00, *p* = 0.02, Total Delays without Language at 6 months in GA: 4.20 ± 0.65 and GB: 6.56 ± 0.85, *p* = 0.03. Total delays at 12 months in GA: 10.06 ± 1.67 and GB: 17.14 ± 1.93, *p* = 0.007. Total Delays without Language at 12 months in GA: 8.58 ± 1.49 and GB: 15.03 ± 1.68, *p* = 0.005.	*ALT* (≥100 IU/L): 6/35 in GA vs. 6/36 in GB, *p* = 0.89. Platelet count (≤100,000/mm^3^): 12/35 in GA vs. 14/36 in GB, *p* = 0.68. Abnormal bilirubin: 5/35 in GA vs. 6/36 in GB, *p* = 0.63. Absolute neutrophil count Grades 3–4: 7/35 in GA vs. 4/36 in GB, *p* = 0.30.
Amir 2010 [25]	Initially, IV GC, 5 mg/kg, every 12 h for 6 weeks. Thereafter, OR VGC 2 daily doses (dose (mg) = body surface area × creatinine clearance (Schwartz equation) × 7) every 12 h were given for the first 6 wks of and then one daily dose up to age 1 year.		Mild hearing loss, 21–45 dB; moderate hearing loss, 46–70 dB; and severe hearing loss, >71 dB.	BSER assessed at birth, at 6 months, and at about 1 year.	A total of 13/23 had hearing defects of various levels after birth compared with 8/21 at ≥1 year.	Formal developmental assessments were not performed as part of the follow-up, but developmental milestones were assessed during the neurological examination at every ambulatory visit.	Once a month up to age 3 months and every 3–4 months thereafter up about to 2 years.	Overall, 4/22 mental retardation.	The main side effects of the treatment were *neutropenia* (12 patients) and *central line infection* (2 patients).
Foulon 2012 [17]	IV GC during 6 wks.		Progressive hearing loss was defined as a worsening of the auditory threshold with 10 dB or more in successive hearing tests. Mild hearing loss 21–40 dBHL. Moderate hearing loss 41–70 dBHL. Severe hearing loss 71–90 dBHL. Very severe hearing loss 91–119 dBHL. Profound hearing loss More than 120 dBHL.	ABR hearing tests were performed within the first month after birth, and on two other occasions in the first year of life. If hearing tests were normal, an annual hearing assessment was performed thereafter.	Normal hearing in two out of six. One out of six had profound hearing loss on both sides that did not change with therapy. One out of six had progression of SNHL and developed a profound bilateral SNHL. One out of six improved from bilateral very severe to bilateral severe hearing loss.	NE			Neutropenia in one out of six.
del Rosal 2012 [18]	OR VGC 32 mg/kg/day. Some also received IV GC prior to VGC, at 12 mg/kg/day.		Hearing was tested by brainstem auditory evoked response (BAER). Results were analyzed considering total evaluable ears and hearing loss degree was categorized as follows: mild hearing loss (21–45 dB), moderate hearing loss (46 70 dB), or severe hearing loss (≥71 dB).	At the time of diagnosis, 6 and 12 months after the beginning of antiviral therapy.	Overall, 11/13 children had hearing defects at baseline compared with 7 at 12 months.	NE			*Neutropenia:* 6/13 patients developed neutropenia. *Transiently raised aminotransferases:* 4/13.
Amir 2013 [26]	Infants were treated with one of the following two protocols: (a) IV GC 5 mg/kg/d for 6 wks followed by OR VGC 17 mg/kg/dose in two daily doses for another 6 wks and then one daily dose until 1 year of age or (b) OR VGC 17 mg/kg/dose in two daily doses for 12 wks and then one daily dose for 9 months.		Defined 25 to 44 dB as mild SNHL; 45 to 69 dB as moderate SNHL; and ≥70 dB as severe SNHL. Improvement was defined as a decrease of ≥10 dB in the auditory threshold on consecutive BERA assessments and a change in category (severe → moderate) between the pretreatment and final assessment.	BSER up to 2 years of life.	Last follow-up: Mild: 2/40 ears; Moderate: 2/40 ears; Severe: 2/40 ears.	NE			*Neutropenia* (<1500/mm^3^): 11/21.
Kimberlin 2015 [10]	OR VGC (16 mg/kg, every 12 h) for 6 months.	OR VGC (16 mg/kg, every 12 h for 6 wks) + placebo.	Defined 21 to 45 dB as mild hearing loss, 46 to 70 dB as moderate hearing loss, and 71 dB or higher as severe hearing loss.	BSER or visual-reinforcement audiometry was performed at 6, 12, and 24 months.	Best ear hearing at 6 months was similar in the two groups (2 and 3 participants, respectively, had improvement; 36 and 37 had no change; and 5 and 3 had worsening; *p* = 0.41). Total ear hearing (hearing in one or both ears that could be evaluated) was more likely to be improved or to remain normal in GA than GB at 12 months (73% vs. 57%, *p* = 0.01) and at 24 months (77% vs. 64%, *p* = 0.04).	Bayley-III.	At 12 and 24 months.	GA had higher Bayley-III language-composite scores at 24 months (*p* = 0.005) and higher receptive communication scale scores at 24 months (*p* = 0.003). All the other components of the Bayley-III assessments trended toward improved outcomes among participants in GA.	*Neutropenia*: 10/47 in GA vs. 13/49 in GB (*p* = 0.64). No significant differences in the rate of other adverse events between the two study groups.
Bilavsky 2015 [27]	Group 1: no hearing impairment at birth, not treated. Group 2: LSV and no hearing impairment, treated. Group 3: LSV and hearing loss, treated. Group 4: asymptomatic cCMV, not treated. IV GC (5 mg/kg) for 6 wks followed by OR VGC (2 daily doses of 17 mg/kg) for 6 wks and then one daily dose until 1 year of age or wo daily doses of OR VGC (17 mg/kg) for 12 wks and then one daily dose until 1 year of age.		Hearing deterioration was defined as an increase of ≥10 dB in the auditory threshold in one or two ears during two consecutive BERA assessments or two behavioral tests resulting in a change in the hearing category, such as from normal to mild, mild to moderate, or moderate to severe hearing loss.	Every 4 to 6 months in the first 4 years of life.	*Hearing deterioration* Group 1: 11/13; Group 2: 0/51; Group 4: 5/52. Group 1 vs. Group 2 (*p* < 0.001) and Group 1 vs. Group 4 (*p* < 0.001). Group 2 vs. Group 4 (*p* = 0.008).	NE			Neutropenia (absolute neutrophil count of ≤1000/mm^3^): in the 76 infants in groups two and three, 22 (28.9%) experienced a total of 30 episodes of neutropenia. None had severe neutropenia (<500 mm^3^). Episodes of neutropenia were only observed during the first three months of treatment, mainly during the first six weeks in infants who started intravenous antiviral treatment. No treatment changes were required, just repeated blood counts.
Bilavsky 2016 [28]	IV GC (5 mg/kg/dose every 12 h for 6 wks) followed by OR VCG (17 mg/kg/dose every 12 h for 6 wks and then one daily dose until 1 year of age). Antiviral treatment was started during the first 4 weeks of life.	OR VCG 17 mg/kg/dose every 12 h for 12 wks then one daily dose until 1 year of age. Antiviral treatment was started during the first 4 weeks of life.	Hearing deterioration/improvement was defined as an increase/decrease of ≥10 dB in the auditory threshold on consecutive hearing assessments and a change in the hearing category. Hearing impairment was detected by the BERA test. Mild hearing loss (25–44 dBHL); moderate hearing loss (45– 69 dBHL); and severe hearing loss (≥70 dBHL).	BSER (in children aged ≤2 years) or the behavioral hearing test (in children aged >2 years) was performed on all children during the neonatal period and at follow-up every 4–6 months until age 4 years.	Of the 77 affected ears at baseline, 50/77 improved, 22/77 remained unchanged and 5/77 deteriorated. Among the 24 infants with abnormal hearing at baseline, 17/24 improved and only 1/24 deteriorated. The deterioration rate among patients with moderate hearing loss at birth (5.6%) was significantly higher compared with patients with normal hearing at birth (0.5%) (*p* = 0.03).	NE			*Neutropenia*: 20/33 in GA vs. 13/33 in GB.
Nishida 2016 [29]	OR VGC (16–32 mg/kg/day) for 6 wks and IV immunoglobulin (300 mg/kg/dose) twice within 2 wks after the initiation of VGC. If clinical symptoms such as hepatitis and ABR worsened or blood CMV loads were higher after treatment for 6 wks than before treatment, OR VGC or IV GC were administered for an additional 6 wks. If GC resistance occurred, patients were treated with foscarnet, starting at 180 mg/kg/day for 2 wks, followed by 90 mg/kg/day for an appropriate period.		Normal development was defined as no sequelae; mild impairment as unilateral hearing dysfunction not requiring hearing aids or other mild sequelae; and severe impairment as severe disabilities, including severe developmental delay, epilepsy requiring treatment with antiepileptic drugs, and bilateral hearing dysfunction requiring hearing aids.	ABR were performed at 3–4, 6, 9, and 12 months of age except for the most severe cases, with follow-up every 3–12 months thereafter.	Of the seven with bilateral ABR abnormalities, one improved to a unilateral ABR abnormality and three recovered to normal. Of the two with unilateral ABR abnormalities before treatment, one recovered to normal. One patient developed late-onset hearing dysfunction.	Kyoto Scale of Psychological Development (normal range 80–110).	At 18–24 months and 3 years of age.	Overall, 4/12 severe impairment (cerebral palsy), 3/12 mild impairment, and 5/12 normal development.	Neutropenia: 7/12; genital bleeding: 1/12; thrombocytopenia 5/5; hepatitis 4/5.
Koyano 2018 [30]	Ten neonates symptomatic at birth received 6 weeks of VGCV (16 mg/kg/dose, twice a day) or ganciclovir GC; 6 mg/kg/dose, twice a day. One of the ten patients received foscarnet, because the blood viral load did not decrease with other medication.		Abnormal wave V (unclear and/or increased latency) at 40 dB.	ABR up to 2 years.	In treated children, in two patients, the hearing impairment disappeared after treatment. Asymptomatic group: late-onset bilateral hearing impairment in 1/26.	WISC-III or -IV or the Kyoto Scale.	For >2 years.	Asymptomatic group: speech delay without hearing impairment in 2/26, autism spectrum disorder in 1/26, and attention deficit–hyperactivity disorder in 1/26.	NE
Pasternak 2018 [31]	IV GC 5 mg/kg die for 6 wks followed by OR VGC 17 mg/kg/dose in two daily doses for another 6 wks and then one daily dose until completion of 12 months of treatment.	OR VGC 17 mg/kg/dose in two daily doses for 12 wks and then one daily dose until completion of 12 months of treatment.	Hearing deterioration or improvement: increase or decrease of ≥10 dB in the auditory threshold on consecutive hearing assessments and change in the hearing category. Mild hearing loss 25–44 dBHL, moderate hearing loss 45–69 dBHL, and severe hearing loss ≥ 70 dBHL.	BSER (≤2 years) or the behavioral hearing test (≥2 years) during the neonatal period and at follow-up every 4–6 months until age 4–5 years.	Of the 80 affected ears at baseline 68.8% improved and only 2.5% deteriorated. Overall, 96.3% of the improved ears returned to normal. Ears with milder hearing loss were more likely to improve with 92.6% improvement vs. 70% from moderate hearing loss and 15.7% from severe hearing loss (*p* < 0.001). No significant difference between GA and GB.	NE			*Neutropenia*: 30 episodes occurred in 19 infants that were observed mainly during the first 3 months of treatment. No statistically different rates of nerutropenia between the two groups.
Ziv 2018 [32]	OR VGC 17 mg/kg/dose in two daily doses for 12 wks, then one daily dose until the age of 1 year. Treatment was started in the first 4 wks of life.		NE			NE			-In total, 46/160 children experienced at least one episode of *neutropenia.* -In total, 12/160 children experienced *anemia*. -No cases of *thrombocytopenia or pancytopenia*. -No cases of *malignancy* (blood or solid).
McCrary 2019 [23]	Children were initially offered VGC at 16 mg/kg for 6 wks. After the 2015 Kimberlin et al. paper demonstrated greater efficacy with a longer treatment course of six months, this approach was offered. Clinically significant worsening of hearing was defined as the occurrence of either (a) 10 dB or greater increase in the minimum response level at both 2 and 4 kHz, (b) 15 dB or greater increase at either frequency, or (c) cochlear implantation.		Clinically significant worsening of hearing was defined as the occurrence of either (a) 10 dB or greater increase in the minimum response level at both 2 and 4 kHz, (b) 15 dB or greater increase at either frequency, or (c) cochlear implantation.	Not scheduled. Patients were followed with regular audiologic assessments, with an average of 5.8 assessments being performed on each study participant.	Overall, 14/16 patients (87.5%, *p*-value < 0.001) were found to have clinically significant worsening of hearing.	NE			NE
Ohyama 2019 [33]	OR VGC 32 mg/kg/day for 6 wks.	OR VGC 32 mg/kg/day for 6 months.	Hearing deterioration or improvement: increase or decrease of ≥20 dB in the wave V threshold of the ABR. Mild hearing dysfunction 31–40 dB, moderate hearing dysfunction 41–60 dB, severe hearing dysfunction 61–90 dB, and most severe hearing dysfunction > 90 dB.	Auditory brainstem response before treatment and at 6 months.	Hearing function improved in 16 (55%) and was maintained in 11 (38%) of 29 abnormal ears; however, deterioration was observed in 2 (7%). In comparison, the hearing function was maintained in 20 (87%) but worsened in 3 (13%) of 23 non-abnormal ears. No statistically significant differences in efficacy between GA and GB (*p* = 1.00); the hearing function was improved or maintained with the same degree in 22 (92%) of 24 abnormal ears in GA and 5 (100%) of 5 abnormal ears in GB. Furthermore, the hearing function was maintained in 14 (88%) of 16 non-abnormal ears in GA and 6 (86%) of 7 non-abnormal ears in GB.	NE			Adverse events: GA: 10/20 and GB: 1/6 (*p* = 0.33). Neutropenia was the most common adverse event in 10/11. Other events included thrombocytopenia 2/11; genital bleeding 1/11, impetigo 1/11, and hypocalcemia 1/11.
Dorfman 2019 [34]	*Symptomatic at birth* Protocol 1 (until 2011): IV GC 5 mg/kg/dose in two daily doses for 6 wks followed by OR VGC 17 mg/kg/dose in two daily doses for 6 wks and then one daily dose until 1 year of age. Protocol 2 (since 2011): OR VGC 17 mg/kg/dose in two daily doses for 12 wks then one daily dose until 1 year of age.	*Asymptomatic at birth* Not treated. If late-onset SNHL was found, the same antiviral treatment as described for symptomatic infants at birth, for a period of 1 year, was given.	Mild hearing loss (25–44 dB); Moderate hearIng loss (45–69 dB); Severe hearing loss (> 70 dB).	BSER (≤2 years) or a behavioral hearing test (>2 years). At birth and every 4–6 months until 5 years.	Of the 45 affected ears in GA, 30 (66.7%) improved and only 2 (4.4%) deteriorated, with most of the improved ears (27/30, 90%) returning to normal. In GB, of the 42 deteriorated ears, 38 (90.5%) improved after at least 1 year of follow-up after late treatment.	NE			*Neutropenia*: 3/66 in GA vs. 1/25 in GB.
Suganuma 2020 [35]	OR VGC for 6 months: initial dose 14–15 mg/kg twice a day and then increased to 16 mg/kg one wk after confirming that there were not adverse effects.		Mild hearing loss: 21–45 dB; Moderate hearing loss: 46–70 dB; Severe hearing loss: ≥71 dB. Improved or worsened hearing: a 20 dB decrease/increase in hearing or changes in at least one category of the wave-V threshold after treatment compared to that at baseline. Unchanged hearing: no change from the wave-V from baseline category, meaning changes were less than 20 dB.	ABR was performed at birth and after 6 months.	Of the 38 ears with impaired hearing at baseline, 9 (23.7%) improved and 29 (76.3%) maintained their hearing status after VGC treatment. Patients with more than moderate at baseline were likely to have improved hearing; eight (88.8%) showed improvement from moderate or severe and only one (11.2%) showed improvement from mild. In total, 5 of 21 sensorineural hearing loss patients (19.2%) had improved hearing function after treatment. In addition, the hearing in 16 sensorineural hearing loss patients (76%) remained unchanged, and the remaining 5 patients with bilateral normal hearing at baseline maintained their hearing during treatment.	NE			*Neutropenia*: 26/26.
Jedlińska-Pijanowska 2020 [19]	IV GC 6 mg/kg every 12 h for 6 wks *or* IV GC 6 mg/kg every 12 h for 3 wks + OR VGC 16 mg/kg every 12 h for 6 months.	OR VGC 16 mg/kg every 12 h for 6 months.	NE			NE			*- Thrombocytopenia* < 100 g/L: GA: 1/60 and GB: 0/38 (*p* > 0.05). - *Cholestasis* (direct bilirubin >1 mg/dL): GA: 5/60 and GB: 0/38 (*p* > 0.05). - *AST*: GA: 45 ±38 U/L and GB: 44 ±31 U/L (*p* > 0.05). - *ALT*: GA: 36 ±30 U/L and GB: 36 ±33 U/L (*p* > 0.05). - *Neutropenia* (<1000 g/L): GA: 15/60 and GB: 10/38 (*p* > 0.05). - *Severe neutropenia* (<500 g/L): GA: 5/15 and GB: 1/10.
Turriziani Colonna 2020 [12]	OR VGC 32 mg/kg/day divided into two daily doses, for a variable number of 6 wk cycles.		ABR: Unilateral or bilateral hypoacusia: mild 21–40 dB; average 41–70 dB; severe 71–90 dB; deep > 90 dB.	Three years.	Overall, 6/35 patients developed SNHL.	Neurocognitive: WPPSI-III, WISC-IV, Leiter-R. Neuropsycological: NEPSY-II, Bell test. Language: bvl_4–12, TFL, Griffiths.	Test were performed according to age in a long-term follow-up.	Cognitive: abnormal in 4/35 patients. Neuropsychological: abnormal in 11/21. Language: pathological in 6/21.	NE
Yang 2021 [36]	IV GC 6 mg/kg for 12 h/time for 6 wks.	OR VGC 16 mg/kg for 12 h/time for 6 wks.	TEOAE: pass or fail. BAEP: mild hearing loss 16–40 dB, moderate to severe hearing loss 41–70 dB, severe hearing loss 71–90 dB, and extremely severe hearing loss > 90 dB.	TEOAE and BAEP during and after treatment.	Normal hearing before and after treatment in GA: 12/24 and GB: 9/24, *p* = 0.112, and GA: 19/24 and GB: 17/24, *p* = 0.376. Mild hearing loss before and after treament in GA: 4/24 and GB: 5/24, *p* = 0.572, and GA: 2/24 GB: 3/24, *p* = 0.637. Moderate to severe hearing loss before and after treament in GA: 3/24 and GB: 4/24, *p* = 0.613, and GA: 1/24 and GB: 3/24, *p* = 0.296. Severe hearing loss before and after treament in GA: 4/24 and GB: 5/24, *p* = 0.572, and GA: 2/24 and GB: 1/24, *p* = 0.637. Extremely severe hearing loss before and after treament in GA: 1/24 and GB 1/24, *p* = 0.817, and GA: 0/24 and GB: 0/24.	NE			*Neutropenia*: 7/24 in GA vs. 8/24 in GB. *Thrombocytopenia*: 0/24 in GA vs. 1/24 in GB.
Wongwathanavikrom 2021 [37]	GC IV for 6 wks.	GC IV 6 wks and OR VGC up to 3–6 months.	The hearing outcomes were classified into normal and hearing loss using the updated American Speech–Language–Hearing Association Criteria.	At 6 and 12 months and at 2–3 years of age.	A statistical analysis of the treatment on hearing outcome was not performed. However, the study found that there was no difference in the hearing outcomes at 6, 12, and 24 months age between the antiviral-treated and untreated groups [hazard ratio (95% CI) of 1.7 (0.6 to 4.9), *p* = 0.308].	Disabilities was diagnosed by pediatricians or pediatric neurologists and defined as impairment in physical, mental, vision, hearing, cognition, communication, developmental, or other conditions that interfere with the patient’s ability to engage in certain actions and requires special helps in some other ways during the patient’s daily life. Global delay development was defined as delay in at least two aspects of development.	ND	A statistical analysis of the treatment on the neurological outcome was not performed. However, patients who received antiviral treatment had a lower proportion of disability than those who did not receive treatment [16.7% versus 47.1%, relative risk ratio of 0.35 (0.12 to 1.06), attributable risk –0.3 (–0.5 to –0.1), *p* = 0.030].	*Anemia*: 9/13 in GA vs. 5/5 in GB. *Neutropenia*: 5/13 in GA vs. 2/5 in GB. *Thrombocytopenia*: 6/15 in GA vs. 2/5 in GB. *Transaminitis*: 5/13 in GA vs. 1/5 in GB.
Leung 2022 [24]	A total of 29/363 infants were prescribed GC only, 228/340 VGC only, and 85/340 both.		NE			NE			Neutropenia: GC 6/29; VGC 39/228; Both 22/85.
Venturini 2023 [20]	OR VGC 16 mg/kg twice a day for 6 wks.	OR VGC 16 mg/kg twice a day for 6 months. + GROUP C (GC): untreated.	Mild hearing loss: 26–40 dB; Moderate hearing loss: 41–60 dB; Severe hearing loss: 61–80 dB; Profound hearing loss: >81 dB. Late hearing loss: deterioration in the degree, in the localization (from unilateral to bilateral), or both and when a child with a normal hearing function at birth developed late hearing loss.	BSER or TEOAE or conditioned play audiometry every 6 months until 3 years of life and then once a year until 6 years.	Late hearing loss: GA: 2/24, GB: 0/26, and GC: 2/48.	NE			*Anemia*: 13/24 in GA vs. 15/26 in GB (*p* = 0.802). *Increase in ALT value*: 25% in GA vs. 30.8% in GB (*p* = 0.650). *Neutropenia*: 5/24 in GA vs. 7/26 in GB (*p* = 0.614).

Table legend. GA (Group A); GB (Group B); NDV (neurodevelopment); GC (ganciclovir); VGC (valganciclovir); OR (orally); IV (intravenously); wks (weeks); BSER (brainstem evoked response); NE (not evaluated); ALT (alanine aminotransferase); TEOAE (transitory evoked otoacoustic emission); ND (not declared); h (hour); ABR (auditory brainstem response); AST (Aspartate aminotransferase); BAEP (brainstem auditory evoked potential).

**Table 5:** Enrolled randomized controlled studies.

Reference	Group A (GA)	Group B (GB)	Outcome		
			Hearing	NDV	Side Effects
Nigro 1994 [15]	GC: 5 mg/kg twice daily for 2 wks IV	GC: 7.5 mg/kg twice daily for 2 wks, and 10 mg/kg three times weekly for 3 months IV	NE	NE	Increased liver enzyme activity: two out of six in GA vs. zero out of six in GB; §. High bilirubin level (>2.5 mg/dL): one out of two in GA vs. zero out of six in GB; § Low platelet count (<90,000/ram 3): one out of six in GA vs. zero out of six in GB; § *Low hemoglobin value* (<9 gm/dL): two out of six in GA vs. zero out of six in GB; §. *Neutropenia* (<1000 cells/mm^3^): zero out of six in GA vs. one out of six in GB §.
Kimberlin 2003 [9]	GC (6 mg/kg per dose IV every 12 h for 6 wks)	No treatment	-A total of 0/25 in GA vs. 7/17 in GB had worsening in hearing at 6 months, *p* < 0.001. -A total of 5/24 in GA vs. 13/19 in GB had worsening of hearing after 1 year, *p* = 0.002.	NE	*ALAT* (≥540 IU/L): 0/40 in GA vs. 0/40 in GB; *p* = 1.00. *Total bilirubin* (according to age and days of life): 11/43 in GA vs. 7/39 in GB; *p* = 0.44. *Thrombocitopenia* (<50.000/mm^3^): 3/45 in GA vs. 2/41 in GB; *p* = 1.00. *Neutropenia* (Grade 3–4): 29/46 in GA vs. 9/43 in GB; *p* < 0.01.
Oliver 2009 [22]	IV GC 12 mg/kg/die in two doses for 6 wks	No treatment	NE	Six wks Personal/Social: 0.5 ± 0.12 in GA (34) vs. 0.78 ± 0.12 in GB (40); *p* = 0.11. Fine motor: 0.21 ± 0.07 in GA (34) vs. 0.28 ± 0.7 in GB (40); *p* = 0.50. Gross motor: 0.09 ± 0.05 in GA (34) vs. 0.18 ± 0.06 in GB (40); *p* = 0.29. Language: 0.71 ± 0.14 in GA (34) vs. 0.83 ± 0.13 in GB (40); *p* = 0.54. Total Delays: 1.5 ± 0.27 in GA (34) vs. 2.05 ± 0.27 in GB (40); *p* = 0.15. Total Delays without Language: 0.79 ± 0.18 in GA (34) vs. 1.23 ± 0.19 in GB (40); *p* = 0.11. Six months Personal/Social: 0.77 ± 0.16 in GA (35) vs. 1.21 ± 0.20 in GB (39); *p* = 0.10. Fine motor: 1.31 ± 0.29 in GA (35) vs. 2.46 ± 0.37 in GB (39); *p* = 0.02. Gross motor: 2.11 ± 0.32 in GA (35) vs. 2.90 ± 0.42 in GB (39); *p* = 0.15. Language: 0.26 ± 0.13 in GA (35) vs. 0.95 ± 0.22 in GB (39); *p* = 0.009. Total Delays: 4.46 ± 0.74.0 in GA (35) vs. 7.51 ± 1.00 in GB (39); *p* = 0.02. Total Delays without Language: 4.20 ± 0.65 in GA (35) vs. 6.56 ± 0.85 in GB (39); *p* = 0.03. Twelve months Personal/Social: 1.28 ± 0.23 in GA (35) vs. 2.22 ± 0.28 in GB (36); *p* = 0.01. Fine motor: 3.31 ± 0.66 in GA (35) vs. 6.19 ± 0.72 in GB (36); *p* = 0.004. Gross-motor: 4.00 ± 0.69 in GA (35) vs. 6.61 ± 0.75 in GB (36); *p* = 0.01. Language: 1.23 ± 0.26 in GA (35) vs. 2.11 ± 0.31 in GB (36); *p* = 0.03. Total Delays: 10.06 ± 1.67 in GA (35) vs. 17.14 ± 1.93 in GB (36); *p* = 0.007. Total Delays without Language: 8.58 ± 1.49 in GA (35) vs. 15.03 ± 1.68 in GB (36); *p* = 0.005.	*ALAT* (≥100 IU/L): 6/35 in GA vs. 6/36 GB; *p* = 0.89. *Platelet count* (≤100,000/mm^3^): 12/35 in GA vs. 14/36 in GB; *p* = 0.68 *Abnormal bilirubin*: 5/35 in GA vs. 6/36 in GB; *p* = 0.63. *Absolute neutrophil count Grade 3–4:* 7/35 in GA vs. 4/36 in GB; *p* = 0.30.
Kimberlin 2015 [10]	OR VGC (16 mg/kg, every 12 h) for 6 months	OR VGC (16 mg/kg, every 12 h for 6 wks) + placebo	Best ear hearing *at 6 months* (worsening): 5/43 in GA vs. 3/43 in GB; *p* = 0.50. Best ear hearing *at 12 months* (worsening): 3/41 in GA vs. 5/40 in GB; *p* = 0.15. Best ear hearing *at 24 months* (worsening): 3/37 in GA vs. 2/31 in GB; *p* = 0.14.	Twelve months Cognitive: 89.6 ± 3.0 in GA (43) vs. 79.5 ± 2.8 in GB (45); *p* = 0.01. Language: 87.6 ± 3.0 in GA (41) vs. 76.8 ± 2.9 in GB (43); *p* = 0.009. Receptive communication: 7.5 ± 0.5 in GA (41) vs. 6.1 ± 0.5 in GB (43); *p* = 0.05. Expressive comunication: 8.0 ± 0.5 in GA (41) vs. 6.5 ± 0.5 in GB (44); *p* = 0.02. Motor: 82.6 ± 3.2 in GA (42) vs. 73.2 ± 3.0 in GB (44); *p* = 0.03. Fine motor: 7.3 ± 0.6 in GA (41) vs. 6.0 ± 0.6 in GB (44); *p* = 0.11. Gross motor: 6.7 ± 0.5 in GA (42) vs. 5.4 ± 0.5 in GB (44); *p* = 0.07. Twenty-four months Cognitive: 84.4 ± 2.6 in GA (42) vs. 76.0 ± 2.6 in GB (41); *p* = 0.02. Language: 84.6 ± 2.9 in GA (41) vs. 72.5 ± 2.9 in GB (41); *p* = 0.004. Receptive communication: 7.3 ± 0.5 in GA (41) vs. 5.2 ± 0.5 in GB (41); *p* = 0.003. Expressive comunication: 7.3 ± 0.5 in GA (41) vs. 5.5 ± 0.5 in GB (41); *p* = 0.02. Motor: 85.5 ± 3.3 in GA (41) vs. 74.1 ± 3.2 in GB (40); *p* = 0.01. Fine-motor: 8.0 ± 0.6 in GA (42) vs. 6.4 ± 0.6 in GB (40); *p* = 0.06. Gross-motor: 7.0 ± 0.5 in GA (42) vs. 5.3 ± 0.5 in GB (40); *p* = 0.02.	*Neutropenia*: 10/47 in GA vs. 13/49 in GB; *p* = 0.64.
Yang 2021 [36]	IV GC 6 mg/kg for 12 h/time for 6 wks	OR VGC 16 mg/kg for 12 h/time for 6 wks	Mild hearing loss after treament: 2/24 in GA vs. 3/24 in GB; *p* = 0.637. Moderate to severe hearing loss after treament: 1/24 in GA vs. 3/24 in GB; *p* = 0.296. Severe hearing loss after treament: 2/24 in GA vs. 1/24 in GB; *p* = 0.637. Extremely severe hearing loss after treament: 0/24 in GA vs. in 0/24 GB; §.	NE	*Neutropenia*: 7/24 in GA vs. 8/24 in GB; *p* = 0.755. *Thrombocytopenia*: 0/24 in GA vs. 1/24 in GB; *p* = 0.312.

Table legend. GA (Group A); GB (Group B); GC (ganciclovir); VGC (valganciclovir); wks (weeks); OR (orally); IV (intravenously); NE (not evaluated); ALT (alanine aminotransferase); § *p*-value not declared.

## Abbreviations

cCMV	congenital cytomegalovirus.
CFS	cerebrospinal fluid.
CNS	central nervous system.
GC	ganciclovir.
IV	intravenously.
LO-SNHL	late-onset sensorineural hearing loss.
MRI	magnetic resonance imaging.
OR	orally.
RCTs	randomized clinical trials.
SGA	small-for-gestational age.
SNHL	sensorineural hearing loss.
VGC	valganciclovir.
